# Effect of educational intervention based on health belief model on nurses’ compliance with standard precautions in preventing needle stick injuries

**DOI:** 10.1186/s12912-023-01347-0

**Published:** 2023-05-25

**Authors:** Navid Alinejad, Mostafa Bijani, Mahmoodreza Malekhosseini, Mahsa Nasrabadi, Pooyan Afzali Harsini, Ali Khani Jeihooni

**Affiliations:** 1 Department of Public Health, School of Health, Fasa University of Medical Sciences, Fasa, Iran; 2 Department of Medical Surgical Nursing, School of Nursing, Fasa University of Medical Sciences, Fasa, Iran; 3 Department of Health Promotion and Aging, School of Health, Shiraz University of Medical Sciences, Shiraz, Iran; 4 Department of Public Health, School of Health, Kermanshah University of Medical Sciences, Kermanshah, Iran; 5 Nutrition Research Center, Department of Public Health, School of Health, Shiraz University of Medical Sciences, Shiraz, Iran

**Keywords:** Health Belief Model (HBM), Nurse, Prevention, Needle Stick

## Abstract

**Background:**

The increasing prevalence of needle stick injuries among nurses and the arising risks double the need to pay attention to improve their knowledge and change their behavior using effective educational models. The present study aimed to investigate the effect of educational intervention based on the health belief model on nurses’ compliance with standard precautions in preventing needle stick injuries.

**Methods:**

This quasi-experimental study was conducted on 110 nurses working in medical training centers in Shiraz and Fasa in 2019. Subjects were selected using a simple sampling method and were randomly divided into two interventions (n = 55) and control (n = 55). The intervention included 7 sessions of 50–55 min. Before and 3 months after the intervention, the health belief model questionnaire was completed by both groups. The data were analyzed using SPSS software version 22 through chi-square, independent, and paired t-tests (P < 0.05).

**Results:**

Based on independent and paired t-tests, there was no significant difference between the control and intervention groups regarding the mean score of health belief model constructs before the intervention. However, there was a significant difference regarding the mentioned scores 3 months after the educational intervention. Based on the paired t-test, the mean score of awareness, perceived sensitivity, perceived severity, perceived benefits, self-efficacy, cues to action, and behavioral performance in the intervention group significantly increased after the educational intervention (P < 0.05). Also, there was a significant decrease in perceived barriers (P < 0.05).

**Conclusion:**

It is recommended to apply the proposed model as an effective and cost-effective method along with other methods in training programs for nurses and other health workers exposed to invasive procedures, contaminated blood, and secretions.

## Background

Needle stick injuries are common and significant occupational hazards for healthcare workers [1]. Needle stick is a term for occupational accidents caused by healthcare workers needles or medicalusing needles, or sharp objects that come into contact with fluids [2]. Due to the nature of their work, healthcare workers face a wide range of chemical, physical, and biological occupational hazards. They are often exposed to injuries caused by needles and sharp tools [3, 4]. Needle stick injury statistics are estimated to be even more common than the rates reported to the Committee on Infection Control. Nurses perform the most hazardous activities because they spend more time with patients than other medical personnel [5]. Many reports show that health workers are at risk of HBV, HCV, and HIV infections because they are exposed to blood and body fluids [6]. Every year, a large number of healthcare workers around the world are infected in this way. Needle stick injuries are caused through the skin and usually the hands are accidental occupational exposures [7–9]. Attention to the consequences of common needle stick injuries is strongly focused on the risk of infection from blood-borne pathogens [10]. Needle stick injuries are the most common occupational hazard for nurses and other healthcare workers. These are the most common way of transmitting viruses or infections, such as HIV and hepatitis, from blood-contaminated needles and sharp objects to healthcare workers [11]. Needle stick Injuries are defined as accidental penetrating skin injuries caused by needles. In developing countries, needle stick injuries are associated with the highest global prevalence of HIV, hepatitis B, and hepatitis C transmission [12]. Occupational exposure exposes health service providers to various infections that threaten the health process [13]. The rate of occupational injuries among healthcare workers due to needle sticks is 19–76% [14]. Approximately 66.7% of nursing staff are exposed to needle and sharp tools injuries during their working hours in the hospital [12]. An injury is an incised wound caused by the penetration of a needle or sharp tool. Each year, more than half a million healthcare workers experience a needle stick injury, increasing exposure to infectious diseases [15]. due to improper disposal and failure to comply with standard precautions [16]. Needle stick, as a type of occupational accident, is rooted in many causes, including a weak safety environment or non-compliance with standard precautions [17]. Among different countries, 3.6% of infections occur in Iran [6]. Hepatitis B is highly infectious and has the heaviest public health burden of all hepatitis viruses. More than 292 million people worldwide are chronically infected and up to 2.4 million in the United States develop silent clinical liver damage after infection. It has surpassed other major infectious diseases (such as HIV, diarrheal disease, malaria, and tuberculosis) as the leading cause of death worldwide. Prevention of transmission is essential [18]. Hepatitis causes about 1.34 million deaths worldwide each year. 1.95% of deaths and complications caused by viral hepatitis are due to chronic hepatitis B and C. 3–5% of Iran’s population is infected with HBV. In 70% of cases, these viral hepatitis strains, highly endemic to sub-Saharan Africa, progress to chronicity, leading to liver cirrhosis and hepatocellular carcinoma, the most common cause of death among HBV and HCV patients. Both HBV and HCV are transmitted through contact with body fluids, especially blood, which are commonly encountered by health care workers through needle stick injuries [19]. One of the confident ways to prevent infections in occupational exposures is compliance with standard precautions. They include hand hygiene, use of personal protective equipment (such as gloves, glasses, and masks), environmental control, waste management and prevention of injuries caused by sharp tools. [20, 21]. Compliance with standard precautions is required as the primary strategy in preventing infections. All hospital staff should have adequate knowledge and skill to implement standard precautions globally [21]. Increased awareness among health care workers and improved needle stick injury reporting systems are necessary to ensure greater protection and early use of prophylaxis after exposure. Implementing safety precautions, safe injection practices and providing engineered safety devices may further reduce the risk [15]. Protective equipment, safety protocols, and tools modification can reduce exposure to hazards [22]. The underreporting of needle stick injuries is still pressing concerns among nursing students [22]. The awareness and empowerment were higher in students who received needle sticks and occupational accident preventive training. Increasing awareness and empowering nursing students through education can reduce the incidence and underreporting of needle sticks [22]. Proper knowledge, safe injection methods, and how to handle needles are important concepts for nurses and healthcare workers [23]. Health and occupational safety in health care workers (HCWs) and compliance with relevant standards and transmission-based precautions are also critical [24]. Surprisingly, the knowledge of nurses and other healthcare workers about needle stick injuries was low. It is important to note that compliance with standard precautions is significantly related to the knowledge. The lower the knowledge, the poorer the compliance, leading to more needle stick incidences. Long-term educational programs are necessary to improve the knowledge of nursing students [25]. The results of different studies showed that the educational intervention based on the health belief model can improve the students’ performance in preventing school injuries [26]. Prevention of needle stick injuries requires situational awareness, which is obtained by using appropriate techniques. Also, the appropriate responses are to conduct risk assessment and group discussions [27]. Choosing the appropriate educational model is the first step in the educational planning. HBM determines the relationship between health beliefs and behavior [28]. This model has six constructs: perceived sensitivity, perceived severity, perceived benefits, perceived barriers, self-efficacy, and cues to action [29]. Despite using personal protective equipment, the risk of self-contamination of health care workers is still high [29]. Providing education on using safety equipment, initial handling of biohazards, and continuous monitoring the staff can improve the nurses’ compliance with standard precautions [30]. Standard precautions are recommended safety measures for healthcare professionals to prevent healthcare-associated infections. Inadequate adherence to these measures can lead to occupational accidents [31]. Education increases health beliefs. Health workers’ awareness regarding the risks of needle stick injuries is insufficient and requires regular training. This ongoing education improves safety cues in health care workers, which subsequently increases the overall level of compliance with standard precautions among nurses. By highlighting interests and removing barriers by proposing workable solutions appropriate knowledge can improve perceived benefits and cues to action, so better performance can be expected [6]. The results showed that HBM constructs are important predictors for compliance with standard precautions among Iranian nurses [32, 33]. According to the health belief model, to adopt preventive actions, people must first feel the risk of the problem (perceived sensitivity), then understand the severity of the various physical and psychological effects of the problem (perceived severity), and take preventive action in case of a positive evaluation of the benefits and the absence of serious barriers. This model has been developed on the basis that it causes people to perceive a health threat, increases their perception of sensitivity and severity of the threat; improves their understanding of benefits of preventive behavior, its perceived barriers and promotes cues to action towards performing health behavior.

### Objective

The present study aimed to investigate the effect of educational intervention based on the health belief model on nurses’ compliance with standard precautions in preventing needle stick injuries.

## Methods

### Design

This quasi-experimental study was conducted on 110 nurses working in Shariati and Valiasr hospitals of Fasa, in the south of Iran, from March 2021 to May 2021.

The sample size for this study was calculated based on Bijani and Bilek et al. study [22, 34]. According to the α = 0.05 and a power of 90% the minimum sample size was set at 45 subjects for each group. To increase power and consider the possibility of loss to follow-up, that number was raised to 60 subjects.

$$n = {\left[ {\frac{{{t_{n - 1,\alpha /2}} + {t_{n - 1,\beta }}}}{d}} \right]^2}{\sigma ^2}$$

**Fig. 1:**
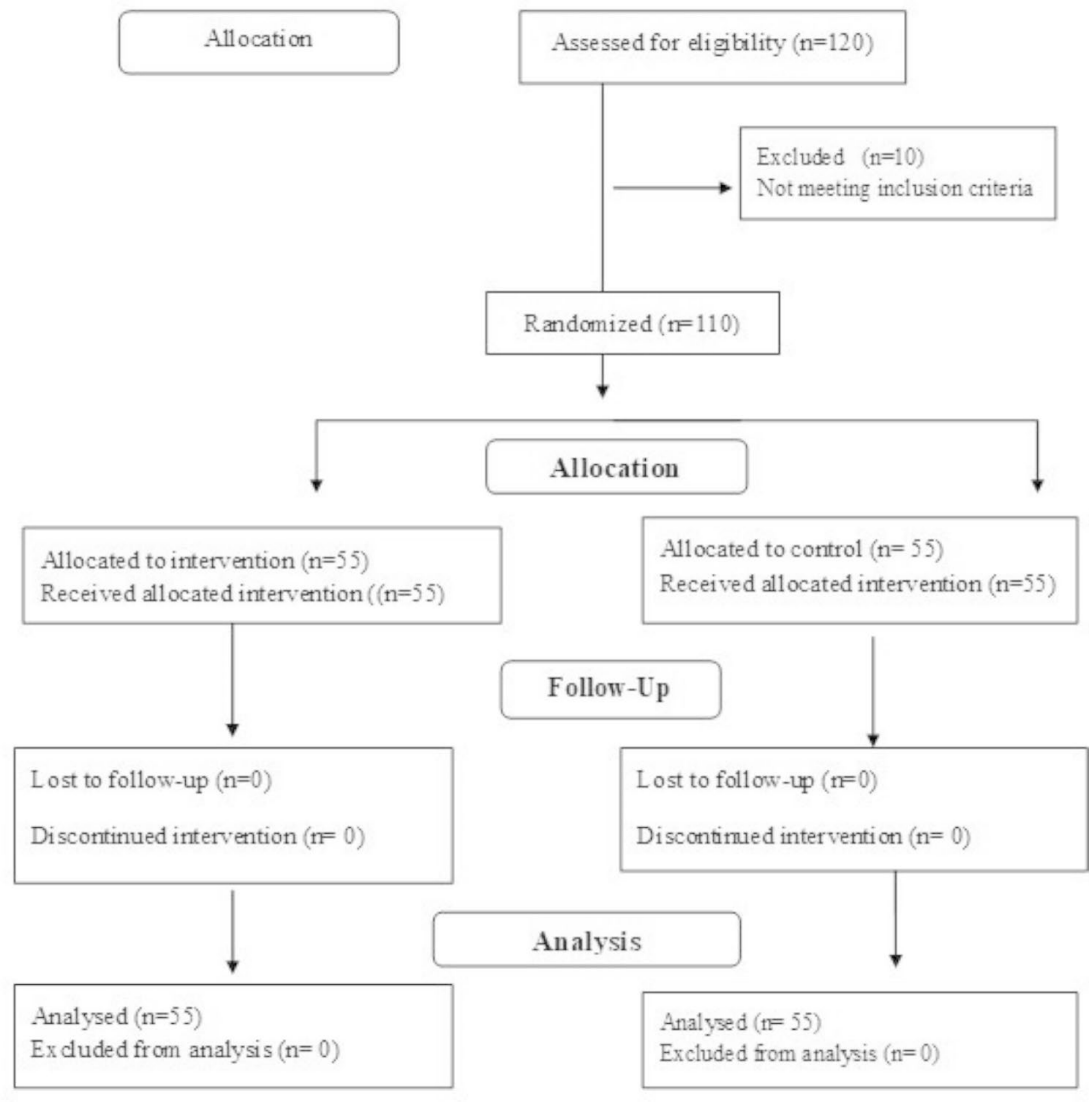
Flow diagram of the participant

The researcher first invited 120 nurses to participate in convenience sampling. Of them, 10 nurses who did not meet the inclusion criteria were excluded. Therefore, the remaining 110 nurses were randomly allocated to two groups, including a control group (group B) and an experimental group (group A). After that, 110 cards were prepared, including 55 cards labeled A and 55 cards labeled B. These 110 cards were then put in an envelope, and each nurse was asked to draw out one card randomly. Each card labeled A and B was the experimental and control group. Figure 1 presents the flow diagram of the participants throughout the study (Fig. 1) .

### Inclusion and exclusion criteria

The inclusion criteria included at least one year of working experience in the hospital, willingness to participate in the study, and not participating in similar training courses. The exclusion criteria were not attending more than two training sessions.

### Data collection

Data were collected using demographic characteristics, health belief model constructs, and performance questionnaires. After receiving permission to enter the research setting, data was collected (from March 2021 to May 2021)by distributing the questionnaires. For this purpose, several liaisons were selected in each hospital to distribute the paper questionnaires. The first author and liaisons gave the questionnaires to eligible nurses. After explaining the methodology and objectives of the study, the participants were asked to complete the questionnaires.

### Demographic characteristics

Demographic characteristics included gender, age, marital status, length of employment, work shift, history of needle stick injury, awareness, and completion of the training course. A score of 1 of 0 was given to the correct and wrong answers, respectively.

### HBM constructs

The health belief model constructs questionnaire contains 39 questions. Three questions about awareness are scored on a 5-point Likert scale (strongly agree score 5 to strongly disagree score 1), the highest score is 15. The lowest score is 3, and a higher score indicates more awareness. Three questions about perceived sensitivity are scored with a 5-point Likert scale (strongly agree score 5 to strongly disagree score 1) with the highest score being 15 and the lowest score 3. A higher score indicates a higher perceived sensitivity. The structure of perceived severity is scored with a 5-point Likert scale (strongly agree score 5 to strongly disagree score 1). The highest score is 15, the lowest score is 3, and a higher score indicates a higher perceived severity. A total of 7 questions related to the structure of perceived barriers are scored on a 5-point Likert scale (strongly agree score 5 to strongly disagree score 1). The highest score is 35, the lowest score is 7, and a higher score indicates a better understanding of the benefits. 5 questions related to the structure of perceived barriers are scored on a 5-point Likert scale (strongly agree score 5 to strongly disagree score 1). The highest score is 25, the lowest score is 5, and a higher score indicates a better understanding of the barriers. A total of 6 questions for the structure of Cues to action are scored on a 5-point Likert scale (strongly agree score 5 to strongly disagree score 1). The highest score is 30 and the lowest score is 6. A higher score indicates better cues to action 5 questions related to the self-efficacy structure, which are scored on a 5-point Likert scale (strongly agree score 5 to strongly disagree score 1), the highest score is 25 and the lowest score is 5, and a higher score indicates better self-efficacy. A total of 7 performance questions related to compliance with standard precautions are scored on a 5-point Likert scale (strongly agree score 5 to strongly disagree score 1). The highest score is 35, and the lowest score is 7, and a higher score indicates better performance in standard precautions.

### Validity and reliability

The validity and reliability of the tools were confirmed in studies by Yousafzai et al. [35]. and Koohsari et al. [36].

To investigate content validity, Content Validity Ratio (CVR) and Content Validity Index (CVI) were used. The experts determined the items’ necessity as ‘necessary’, ‘useful but not necessary’, and ‘not necessary’ considering CVR. In doing so, we collected 15 experts (5 nurses with bachelor’s degrees in nursing and 10 nursing professors with experience in tool design and validation) opinions, and values greater than 0.49 were considered acceptable based on the Lawshe Table [37]. The CVR of 10 items was higher than 0.49. Regarding CVI, the 15 experts were requested to evaluate the items regarding relevance, clarity, and simplicity. In this respect, scores above 0.79 were considered acceptable. Based on the results, the CVI of 10 items was higher than 0.79 [37]. Finally, the reliability of the questionnaire was assessed using the test-retest method. In doing so, the questionnaire was given to 100 nurses in two stages at a two-week interval. The intra-class correlation coefficient (ICC) of the questionnaire was found to be 0.95, which indicates the appropriate internal consistency of this questionnaire.

### Intervention

Before the educational intervention, both experimental and control groups completed the mentioned questionnaires. Then, based on the pre-test results, an educational intervention was implemented for the experimental group through seven 50–55 min training sessions using lectures, question and answer, group discussions, practical demonstrations, video clips, and PowerPoint. These sessions were held once a week. The training were scheduled on free days and were reminded through SMS and telephone. A WhatsApp group was formed and training booklets were provided at the end. Educational and motivational messages and videos were sent weekly to the WhatsApp group. The participants of the intervention group could communicate with other participants to increase their experience and skills, and they could share their information with others. Three months after the educational intervention, both groups completed the questionnaires. The content of the training sessions was as follows:

The first session deals with perceived severity, perceived sensitivity; definitions, and generalities, introducing the level of risk and people at risk by presenting statistics related to needle sticks, contaminated sources, complications, diseases, physical risks, and mental and psychological consequences; the severity of the injuries caused by the needle stick.

The second session deals with perceived barriers, perceived benefits, the cause of needle stick injuries, the effective factors in their occurrence, the high costs of treatment and unemployment and its consequences; the benefits of compliance with standard precautions and reducing stress, increasing job satisfaction, the importance of the safety of health staff, availability of personal protective equipment.

The third session deals with creating self-efficacy, actions before and after exposure to a needle stick, and a practical demonstration of how to put on and take off personal protective equipment.

The fourth session deals with cues to action, the needle stick injury protocol, standard precautions for needle sticks, injury prevention and managed care guidelines, cues to injection safety, effective strategies to reduce risky behavior, and safety guidelines.

The fifth session deals with improving the performance regarding needle stick injuries, how to collect, store, transfer, and dispose of contaminated and hazardous waste, the procedure for dealing with sharp objects, and changing behavior and social attitude.

The sixth and seventh sessions summarize the previous sessions, including definitions, causes and effects, benefits, preventive measures, and post-exposure measures, solutions, instructions, and principles of personal safety.

## Ethical consideration

All the participants gave written informed consent to participate in the study. The present study was conducted in terms of the principles of the revised Declaration of Helsinki, which is a statement of ethical principles that directs physicians and other participants in medical research involving human subjects. The participants were assured about their anonymity and confidentiality of their information. Moreover, the study was approved by the Institutional Research Ethics Committee of Fasa University of Medical Sciences, Fasa, Iran (Ethical code: IR.FUMS.REC.1397. 082 ).

### Data analysis

The data were analyzed using SPSS version 22 software through chi-square, independent, and paired t-tests (P < 0.05). Kolmogorov-Smirnov test showed that the data were normally distributed (P < 0.05).

## Results

This study was conducted on 110 nurses. The experimental and control groups’ mean ages were 39.88 ± 4.12 and 40.66 ± 4.04 years, respectively.

The chi-square test showed no significant difference between the two groups regarding gender, marital status, length of employment, work shift, history of needle stick injury, and completion of needle stick training course (Table 1).

**Table 1:** Demographic characteristics of the studied subjects

Variable		Experimental Group		Control Group		P-Value
		Number	Percentage	Number	Percentage	
Gender	Male	16	32	20	33.33	0.194
	Female	34	68	40	67.66	
Marital status	single	11	22	14	33.23	0.202
	married	39	78	46	67.76	
Length of employment	1–5 years old 6–10 years old ≥ 11 years old	7 12 31	14 24 62	9 16 35	15 67.26 33.58	0.178
Work Shift	1–2 shifts ≥3 shift	27 23	54 46	28 32	67.46 33.53	0.181
History of Needle stick injury	yes no	22 28	44 56	31 29	67.51 33.48	0.084
Needlestick training course	yes no	16 34	32 68	25 35	67.41 33.58	0.102

The independent t-test showed a significant difference between the two groups regarding the mean score of awareness, perceived sensitivity, perceived severity, perceived benefits, perceived barriers, perceived self-efficacy, cues to action, and performance before the educational intervention. However, the two groups had a significant difference regarding the mentioned variables three months after the educational intervention. Also, based on the paired t-test, the mean score of awareness, perceived sensitivity, perceived severity, perceived benefits, perceived self-efficacy, cues to action, and performance significantly increased in the experimental group. In the control group, the mean score of awareness, perceived sensitivity, perceived severity, perceived benefits, perceived barriers, perceived self-efficacy, cues to action, and performance did not change significantly. (Table 2).

**Table 2:** Mean score of HBM constructs and performance

Variables	Group	Before the intervention M ± SD	3 months after the intervention M ± SD	p-value*
**Awareness**	Experimental	7.21 ± 1.43	12.79 ± 1.88	0.001
	Control	7.76 ± 1.58	8.36 ± 1.68	0.226
	P-Value**	0.269	0.001	
**Perceived sensitivity**	Experimental	7.25 ± 1.04	12.78 ± 1.55	0.001
	Control	6.99 ± 1.02	7.90 ± 1.22	0.147
	P-Value**	0.180	0.001	
**Perceived severity**	Experimental	6.84 ± 0.09	13.09 ± 1.12	0.001
	Control	7.21 ± 1.03	8.14 ± 1.07	0.155
	P-Value**	0.195	0.001	
**Perceived benefits**	Experimental	10.18 ± 2.14	20.33 ± 2.47	0.001
	Control	11.33 ± 2.10	12.69 ± 2.34	0.139
	P-Value**	0.146	0.001	
**Perceived barriers**	Experimental	27.17 ± 2.96	10.12 ± 2.75	0.001
	Control	25.62 ± 2.87	23.40 ± 2.52	0.133
	P-Value**	0.128	0.001	
**Self-efficacy**	Experimental	11.14 ± 1.16	22.14 ± 1.65	0.001
	Control	12.06 ± 1.26	13.12 ± 1.29	0.145
	P-Value**	0.159	0.001	
**Cues to action**	Experimental	12.46 ± 2.33	26.27 ± 2.64	0.001
	Control	12.96 ± 2.92	13.98 ± 2.90	0.186
	P-Value**	0.309	0.001	
**Performance**	Experimental	19.20 ± 2.48	30.83 ± 2.74	0.001
	Control	18.94 ± 2.51	19.95 ± 2.54	0.162
	P-Value**	0.276	0.001	

* Paired t test

** Independent T-test

## Discussion

The present study aimed to investigate the effect of educational intervention based on the health belief model on nurses’ compliance with standard precautions in preventing needle stick injuries. This study was conducted on 110 nurses. The experimental and control groups’ mean ages were 39.88 ± 4.12 and 40.66 ± 4.04 years, respectively.

The independent t-test showed a significant difference between the two groups regarding the mean score of awareness, perceived sensitivity, perceived severity, perceived benefits, perceived barriers, perceived self-efficacy, cues to action, and performance before the educational intervention. However, the two groups had a significant difference regarding the mentioned variables three months after the educational intervention. Also, based on the paired t-test, the mean score of awareness, perceived sensitivity, perceived severity, perceived benefits, perceived self-efficacy, cues to action, and performance significantly increased in the experimental group. In the control group, the mean score of awareness, perceived sensitivity, perceived severity, perceived benefits, perceived barriers, perceived self-efficacy, cues to action, and performance did not show significant changes.

The results of a study by Sediq et al. [38] showed that training significantly improves perceived sensitivity, which was consistent with the results of our study. A study by Khodisiave et al. [20] demonstrated that the level of awareness about standard precautions for infection control was poor, while the level of practice was moderate. There was a significant relationship between training and the constructs of perceived benefits, cues to action, perceived sensitivity, and perceived self-efficacy. In addition, perceived benefits had a significant relationship with awareness, which was also consistent with the results of our study.

The results of a study by Ramezani [33] exhibited a significant difference of awareness in different target groups before and after exposure. There was a significant relationship between health motivation and standard precautions. There was also a significant relationship between self-efficacy and nurses’ motivation. Timely cues to action were the strongest predictors of health motivation, and perceived threat was the weakest predictor. In the present study, perceived barriers were one of the important predictors of preventive behaviors and had an inverse relationship with preventive behaviors. Perceived sensitivity and severity create motivation for action, and integrating perceived benefits and barriers provides a tool for action. Therefore, the stronger the perceived sensitivity, the stronger the perceived severity and benefits. Also, the weaker the perceived barriers, the more likely the adoption of preventive health measures. In a study by Sedigh et al., there was a significant relationship between perceived sensitivity and severity with preventive behavior [38]. The results of a study by Suksatan et al. showed that increasing the knowledge of healthcare personnel regarding reducing the incidence of nosocomial infection could reduce the hospitalization rate [39].

Based on the results of a study by Saadeh et al. [40]. The highest number of needle stick injuries were among nurses (39.7%), followed by service workers (36.3%), doctors (10.4%), other staff (7.4%), and laboratory technicians (5.9%). Education among high-risk groups and standard precautions should be implemented to reduce needle stick injuries. Another study by Khodisiave et al. [41] showed that only 5.7% of nurses have good knowledge about standard preventive measures.

Based on the results, there was no significant difference between the two groups regarding the mean score of the HBM constructs before the intervention, indicating the homogeneity of the participants in the two groups. However, the mean awareness score in the experimental group significantly increased after the intervention, indicating the e of educational intervention. Based on the results of various studies, nurses’ knowledge of needle stick injury preventive behaviors is weak [38], [42, 43].

The results of a meta-analysis by Tekalign et al. [44] unraveled that the knowledge and uptake of post-exposure prophylaxis, one of the best approaches to deal with HIV/AIDS transmission, is significantly low. Therefore, healthcare organizations should work on strategies to increase knowledge and uptake of post-exposure prevention among healthcare providers. In a study by Mehravar A et al. [6], nurses’ overall compliance with standard precautions (SP) was moderate. Perceived sensitivity, perceived severity, and perceived benefits had the highest score, respectively, indicating that emergency department nurses take their health status seriously. However, improving perceived barriers, perceived self-efficacy, cues to action and knowledge requires more attention and education. Work experience and education were important factors in overall compliance with precautions among nurses. Health belief constructs showed a positive correlation with standard precautions, and there was a positive relationship between education and compliance with precautions.

The mean of compliance with precautions in nurses with continued education was higher than those without training, which was not statistically significant. Other variables had no significant correlation with compliance with standard precautions. In a similar study, al-Hazmi et al. emphasized the effect of perceived severity, perceived sensitivity, perceived benefit, and cues to action on health workers’ self-efficacy in compliance with standard precautions. This study suggests knowledge as the main solution to increase performance among nurses as a low-cost and direct method.

A study by Rey-Merchán MdC et al. [45] showed that the higher the professional age, the higher the probability of an accident and its severity. This was inconsistent with the results of a study by Mehravar A et al. [6].

In terms of gender, men are exposed to more serious incidents compared to women. In a study by Yasmeen MS et al., continuous standard precautions training showed a significant relationship with knowledge. The overall knowledge of female nurses regarding standard precautions was low. The results of this study showed the need to continue intensive and in-service training sessions on infection control using innovative approaches [46], which is consistent with the results of a study by Sodhi et al. A multifaceted approach of continuous training and feedback programs should be adopted to improve awareness and compliance with infection control practices.

In a study by Adola SG, ineffective training courses led to nurses’ weak knowledge of preventive behaviors. Appropriate knowledge and awareness are one of the basic and necessary principles for changing behavior, which hinges upon effective educational intervention. On average, 83% of participants were aware of how infectious diseases are transmitted, while 96.6% of people with infectious diseases responded that transmission-based precautions help control infection. Also, 98.9% of respondents stated that compliance with standard precautions is vital in infection control. The responding health care workers’ knowledge, attitude and overall performance after training was acceptable [47].

A study revealed that nurses not trained in occupational hazard prevention were twice as likely to suffer from sharp tool injuries. Also, healthcare workers working more than 40 h per week were almost four times more likely to be exposed to the risk of serious injury [47].

A cross-sectional study by Okeke et al. [48] showed a significant relationship between hepatitis B vaccination and needle stick injury. Profession and demonstration of relevant standard precautions positively influenced the occurrence of needle stick injury and HBV vaccination, while blood vaccine-preventable knowledge also influenced HBV vaccination status. Written policies on HBV vaccination and needle stick injury prevention and management, implementing them to health care principles, adequate education, displaying standard precautions, and free HBV vaccination are recommended.

The study of Onubogu, et al., and Shamk, et al. [49, 50] showed that the present study’s mean score was low in all areas related to occupational hazards among nurses. The present study concluded that the overall assessment of nurses’ job risks is low. This study recommends conducting controversial health education courses. It is necessary to increase health awareness among nurses, especially new staff in primary health care centers.

Based on a study by Gebreeyessus [51], cues to action and self-efficacy had the greatest relationship with health in nurses. Changes in interventions and compliance with standard precautions predicted positive results. It was concluded that a prerequisite for increasing the perceived sensitivity is increasing awareness and knowledge about the behavior. Also, pre-employment and in-service trainings significantly affect behavior during the confrontation. There is a positive relationship between risk perception and standard precautions. In this regard, the results of a study by Bilek Ö et al. [22] demonstrated that awareness and empowerment were higher in students who received occupational accident and needle stick injuries prevention training. Also, the results of a similar study by Ilıman AY et al. [52] proved that waste management knowledge and skills are important for students before clinical practice for evaluating and developing safe injection practices. It was found that 40% of injuries occurred during blood sugar measurement, 25% during bleeding, and 26.7% during injection. While 31.7% of injuries occurred during needle recapping, 23.3% were experienced when removing the needle from the patient. It was also found that the use of the sharp needle box for medical waste was low and only 23 (38.3%) of the injured students used it as a precaution. Compliance with standard precautions for infection control among prehospital emergency personnel can be improved by enhancing the perceived benefits, perceived sensitivity, perceived self-efficacy, and cues to action [20].

It may be suggested that studies be conducted on different strategies in the safe management of waste from healthcare activities to investigate their impact on needle stick injuries among students [22]. The preventive measures should be adapted to the profile of nurses in the most vulnerable department [43]. Based on the results of a study by Tawalbeh et al. [53], the lack of effective training courses is the cause of the weak knowledge and awareness of nurses in preventive behaviors. One of the basic and necessary principles for changing behavior is possessing the desired knowledge and awareness. The use of different educational methods, including group discussions, problem-solving thinking, role-playing, brainstorming, and taking advantage of peer education in this study were educational strengths that significantly impacted raising nurses’ awareness. The results of this study regarding increasing nurses’ awareness were consistent with the results of other studies [13, 32, 33, 37, 44, 45, 54].

## Limitations

This study was conducted in the south of Iran. Therefore, due to the different economic, social, and cultural conditions, it is necessary to conduct this study in other countries, and it is also recommended that this study be conducted in nursing students as one of the groups at risk of occupational exposure to needle sticks injuries should be investigated.

## Conclusion

It is recommended to apply the proposed model as an effective and cost-effective method and other methods in training programs for nurses and other health workers exposed to invasive procedures, contaminated blood, and secretions. It is also suggested that this study be carried out on other healthcare workers and units with a larger sample size.

## Data Availability

The datasets used and/or analyzed during the current study can be made available by the corresponding author on reasonable request.

## Abbreviations

HBM: Health Belief Model
HCWs: Health Care Workers
